# FMRFamide-like peptides (FaLPs) - an overview of diverse physiological roles in insects and other arthropods

**DOI:** 10.7150/ijbs.106382

**Published:** 2025-03-31

**Authors:** Szymon Chowański, Jan Lubawy, Joanna Pacholska-Bogalska, Lapo Ragionieri, Arkadiusz Urbański, Monika Szymczak-Cendlak, Karolina Walkowiak-Nowicka, Paweł Marciniak

**Affiliations:** 1 Department of Animal Physiology and Developmental Biology, Adam Mickiewicz University, Poznań, Poland, Uniwersytetu Poznańskiego 6 Str., 61-614 Poznań, Poland.; 2 Competence Centre for Plant Health, Free University of Bozen-Bolzano, Bozen-Bolzano, Italy.

**Keywords:** insects, neuropeptides, GPCR, muscle contraction, physiology, mass spectrometry, -RFamide

## Abstract

FMRFamide-like peptides (FaLPs) are neuropeptides that play a pivotal role in regulating various physiological processes in insects and other arthropods including behaviour, reproduction, and homeostasis. FaLPs mostly act through G-protein coupled receptor and influence muscle activity by modulating Ca^2+^ influx. Historically, the function described for these neuropeptides was primarily associated with myostimulatory activity. After more than three decades of research, it is now well established that FaLPs are implicated in the regulation of circadian rhythms, affecting locomotor activity and phase changes in response to environmental cues. During reproduction, FaLPs influence contractile activity in both the male and female reproductive systems. They also participate in physiological processes such as diapause induction, sleep modulation, and flight regulation in insects. Similarly, in crustaceans, FaLPs regulate the circulatory system, stomatogastric nervous system, and muscle contractions. Nowadays, it is also known how the physiological properties of FaLPs in arthropods share similarities with mammalian RFamide peptides, which are involved in a wide range of functions, including muscle contraction, feeding, reproduction, and stress responses, mediated through various RFamide receptors. Therefore, summarizing the investigated physiological functions in arthropods may be relevant also for future research aiming to test their activity in other organisms such as mammalians.

## Introduction

Neuropeptides play a pivotal role in regulating nearly all physiological processes in various arthropods by acting as neurotransmitters, neuromodulators, or (neuro)hormones. Among these peptides, FMRFamide-like peptides (FaLPs) are a significant group known for their multidirectional influence on arthropod physiology. The term FMRFamide refers to a sequence of four amino acids (Phe-Met-Arg-Phe-NH_2_) initially identified as a cardioacceleratory agent in molluscs 1. When searching the literature, especially from the first decade of this century, readers can be slightly puzzled by the two terms used interchangeably to describe this family of peptides, especially in insects. In insects, FMRFa itself has not been identified, the FaLPs are *N-*terminus extended FMRFamides (Table 1). Prior to the recommendation of Coast and Schooley on a consensus insect peptide nomenclature 2, other families of peptides with similar *C*-terminus sequences such as -FLRFa (myosuppressins, MS), -HM/LRFa/HLRFa (sulfakinins, SK), -RP/VRFa (neuropetides F; NPF), -RLRFa (short neuropeptide F; sNPF), were often described as FMRFamide-related peptides (FaRPs) or FMRFamide-like peptides (FaLPs). Nowadays, these peptides are assigned to separate families because they are encoded by different genes and activate distinct receptors 2. Hence, this review will focus on the FaLPs as defined by Coast and Schooley and which activate the FMRFa receptors. In other subphyla such as arachnids and crustaceans the nomenclature seems to be the same, however in some studies based on mainly on mass spectrometry FaLPs may still embrace sNPF, MS and SK 2, 3.

The physiological properties of FaLPs have been studied in various arthropod species. However, the majority of studies on FaLPs have involved mainly pancrustaceans with special emphasis on insects, and crustaceans (Table 3). Since, there is only a handful of studies on FaLPs in myriapods and chelicerates, we only briefly include these groups in our review.

First research performed on insects and crustaceans revealed at that time that they are myoactive neurohormones that show species and tissue specificity, causing both inhibition and stimulation of heart and visceral organ contraction 4-6.

The influence of FaLPs extends beyond muscle contraction to encompass broader aspects of insect physiology, including the regulation of circadian rhythms, feeding behaviour, and reproductive functions 7. Their widespread distribution and coexpression with other neuropeptides, such as sulfakinins 8, suggest a multifaceted role in the regulation of insect physiology, making them a subject of interest for understanding the intricate network of peptidergic signalling in invertebrates. Furthermore, FaLPs in crustaceans and insects can be considered as analogues (similar function, but different origin) or homologues (common origin, but possibly different functions) of mammalian RFamide neuropeptides. At present it is difficult to decide this issue unequivocally. Thus, knowledge about FaLPs biology can be used to better understand the mechanism of action of neuropeptides in general, not only in insects. These findings may be useful in the future in translational research, e.g., in the search for new solutions for the treatment of animal and even human health disorders.

Until now only few, excellent reviews were published on the influence of FaLPs (designated sometimes as FaRPs) on various aspects of insect physiology 9-12. However, they were published several years ago and focused not only on FaLPs but also on other RFamides (myossupressins, sulfakinins, short neuropeptides F). For this reason, here, we summarize the current knowledge exclusively about FMRFa-like peptides (FaLPs), including their biosynthesis, mode of action, and role as crucial components of insect physiology regulation, with their effects permeating through various systems and processes. Moreover, we compare the properties of insect FaLPs with crustaceans ones, showing their conserved role as myoactive factors which directly or indirectly influence circulatory system and regulate feeding. Finally, we compare the known function in insects with those known for RFamides in mammalians and indicate possible similarities in regulation of chosen physiological processes.

## Identification and precursor structure

### FaLPS in insects

Neuropeptide precursors can be separated into single-copy and multi-copy precursors. The latter group, which includes FaLP precursors, contains a variable number of para-copies of the same neuropeptide family, typically activating the same G protein-coupled receptor (GPCR), with some exceptions, such as pyrokinin precursors, which activate different receptors 13. Generally, each neuropeptide family contains a typical *C*-terminal consensus sequence of five amino acids, which may vary slightly between species in the case of a single copy 14 but also within the same genus for multi-copy precursors. Usually, the number of para-copies is consistent within the same group, but in some instances, it can vary dramatically within the same genus 15.

FMRFamide was originally identified in the sunray venus clam *Macrocallista nimbosa* by Edman degradation 1. In insects, the first FaLP neuropeptide was characterized in *Drosophila melanogaster* 16, 17. Subsequently, these peptides have been identified in nearly all invertebrates and are now recognized as one of the most widely distributed neuropeptide families among arthropods and metazoans. Thus far, only *N*-terminally extended FaLPs have been identified, mainly by mass spectrometry.

**Figure 1:**
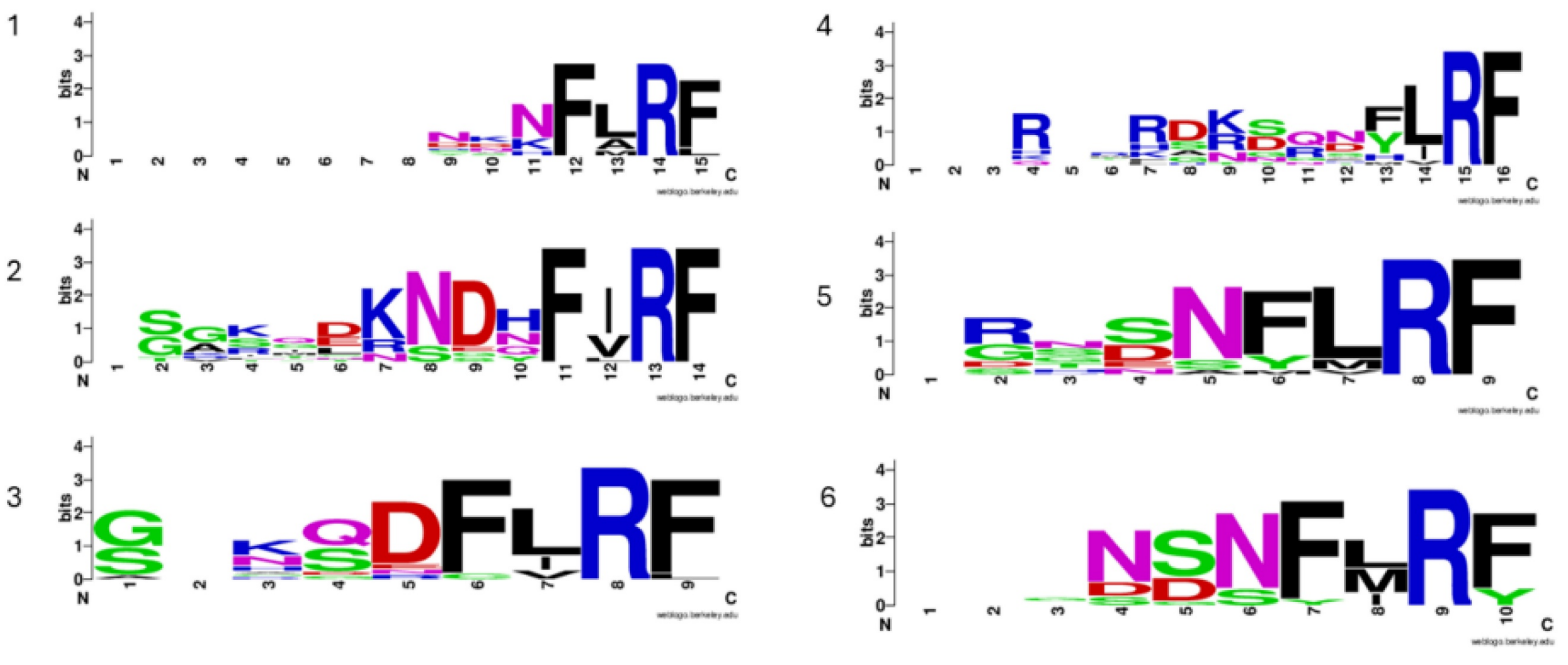
Sequence conservation of the six putative bioactive FaLPs peptides (1-6) in Coleoptera. All precursors were aligned using MAFFT and manually reviewed to correctly align cleavage sites. In the weblogo only putative bioactive peptides with amidated *C*-terminal were included. Species included are: *Aethina tumida* XP_019869081.1; *Aleochara curtula* (SRR921563); *Anoplophora glabripennis* (XP_023310731.1); *Aquatica sp.* (AQULA_011885-PA); *Dendroctonus ponderosae* (XP_019753890.1); *Coccinella septempunctata* (GCA_907165205.1); *Harmonia axyridis* (GCA_914767665.1); *Hycleus* sp. (Hpha006160); *Hypothenemus hampei* (GCA_013372445.1); *Ignelater luminosus* (ILUMI_09630-PA); *Leptinotarsa decemlineata* (XP_023026284.1); *Nicrophorus vespilloides* (XP_017784347.1); *Oryctes borbonicus* (GCA_902654985.2); *Photinus pyralis* (PPYR_06929-PA); *Pogonus chalceus* (JU434250.1); *Tenebrio molitor* (ON110508); *Tribolium castaneum* (GCA_031307605.1).

Subsequent investigations showed that FMRFamide precursors exhibit a variable number of para-copies across taxa (Table 1), often accompanied by slightly different *N*-terminal consensus motifs. In hemimetabolous insects, the number of para-copies varies both across and within groups: in the silverfish *Thermobia domestica* (n = 12; 18; in *Periplaneta americana* (n = 24; 19; in *Zootermopsis nevadensis* (n = 10; 20; in *Schistocerca gregaria* (n = 6; 21; in *Locusta migratoria* (n = 5; 22; in *Carausius morosus* (n = 8; 23; in *Rhodnius prolixus* (n = 1; 24; and in *Acyrthosiphon pisum* (n = 4; 25. In holometabola insects, there is a general reduction in the number of para-copies with *Apis mellifera* (n = 6; 26; *Bombus terrestris* (n = 6; 27; *Manduca sexta* (n = 6; 28; *Bombyx mori* (n = 4; 29; *Spodoptera frugiperda* (n = 5; 29; *Galleria mellonella* (n = 5; 30; *Carabus violaceus* (n = 6; 31; *Hylobius abietis* (n = 6; 32; *Tribolium castaneum* (n = 6; 33; *Tenebrio molitor* (n =6; 34, and *Zophobas atratus* (n = 6; 34. In a recent investigation of the neuropeptidome in Coleoptera, Veenstra 33 reported a variable number of FaLPs para-copies ranging from four to six. Moreover, aligning homologous sequences among different species and groups is difficult both with automatic approaches and with manual editing of the alignments (Figure 1).

Despite the considerable increase in the number of species sequenced using high-throughput techniques in recent years, only relatively few studies have confirmed the peptide sequences of FaLPs neuropeptide family across the central nervous system (CNS) of insects using mass spectrometer such as MALDI (Matrix-Assisted Laser Desorption/Ionization) and/or Orbitrap 15, 21, 23, 25, 31, 32, 34-43.

Collectively, these studies have provided crucial insight into both the localization of neuropeptide neurons in the CNS and the processing of precursors. Historically, the distribution of FaLPs in the CNS was investigated using immunohistochemistry (IHC). However, IHC has limitations for neuropeptide families such as FaLPs since several neuropeptide families share a similar *C*-terminal consensus sequence (-RFamides). These include FaLPs and the above-mentioned MS, SK, sNPF, and NPF families. Indeed, it is likely that the antisera produced against RFamide peptides exhibit cross-reactivity among all these neuropeptide families in the same tissue. Studies employing a combination of IHC and direct tissue profiling initially reported a tagmata-specific distribution of neuropeptides 44. FaLPs are predominantly expressed in posterolateral cells (PLCs) of the thoracic ganglia, with axons projecting into neurohemal organs, the thoracic perisympathetic organs (tPSO), in which mature and bioactive peptides are likely to be stored and released. In most insects, MALDI direct tissue profiling of tPSO contains almost only ion signals of FMRFamide-like peptides. Furthermore, Liessem et al 23 demonstrated, through direct tissue profiling of 16 single PLCs, that the ion intensity of the different para-copies varies considerably, but the pattern remains consistent across different preparations, indicating no alternative processing of these neuropeptide precursors, as was reported for pyrokinins (PKs) 31, 34, 45. FaLPs have also been identified in the cerebral ganglion of *P. americana*, particularly at higher concentrations in the antennal nerve and at lower concentrations in the antennal lobes 42. More recently, using mass spectrometry imaging (MSI), the distribution of FMRFamide-like peptides was shown in sections of the major neurohemal organ of insects, which is connected to the cerebral ganglion and gnathal ganglion, the retrocerebral complex (RCC) of *P. americana* 19. Although, the ion signals of these peptides in RCC are of relatively low intensity compared to those in tPSO, MSI effectively distinguished the distribution of these peptides from other RFamide peptides, such as MS, which is typically abundant in RCC. Additionally, MSI single spectra confirmed a similar pattern of ion signals matching the FMRFamide-like peptide para-copies observed in PLCs, suggesting identical processing of all para-copies in the tPSO as well in the RCC of the analysed insects. Another crucial aspect that can be investigated through direct tissue profiling of tissues abundant in FaLPs is the processing of precursors and postprocessing modifications such as pyroglutamate, which are important for preserving the structure and stability of a few peptides. In *C. morosus*, partial use of internal cleavage sites and unusual cleavage sites of the signal peptide have been reported in the FaLPs precursor 23. The former case occurs in those multicopy precursors when a single monobasic cleavage site, such as a single Arg residue (IFG-RGK), is situated between two consecutive potentially bioactive peptides. The latter could result in the production of either a longer precursor and/or spacer peptide after the signal peptide or an *N*-terminal extended bioactive neuropeptide. Similar occurrences have also been documented for other neuropeptide precursors, such as pheromone biosynthesis activating neuropeptide (PBAN), in moths 46. Incomplete processing of the FaLPs precursor was also observed in Coleoptera. It is noteworthy that in Coleoptera species such as *Carabus violaceus*, the PLCs do not project into a single median tPSO but instead project through transverse nerves into two separated lateral tPSOs posterior to the ganglia 31. Similar thoracic neurohemal organs are found in tenebrionid species such as *T. molitor* and *Z. atratus* 47. Mass spectra of the tPSO organs of *C. violaceus* (Carabidae), *H. abietis* (Curculionidae), and *Z. atratus* (Tenebrionidae) revealed *N*-terminal extended forms, likely resulting from a less efficient cleavage at the Arg residue 31, 32, 34. Currently, it is unclear whether these forms exhibit distinct bioactivity, but it is important to highlight that the combination of IHC and mass spectrometry (especially MSI) is capable of producing the most comprehensive information about both the site of expression and processing of neuropeptides.

### FaLPs in Crustacea

Among crustaceans, FaLPs are localized in both the central and peripheral nervous systems, playing a critical role in regulating several physiological processes. Until 2003, twelve crustacean FaLPs had been identified, mostly in decapods such as *Callinectes sapidus* 48, *Homarus americanus* 49, *Cancer borealis* 50, *Procambarus clarkii* 51, and *Macrobrachium rosenbergii* 52. These neuropeptides were identified in several tissues throughout the nervous systems of different species, including the pericardial organs, the stomatogastric nervous system, and the eyestalk. In the following years, this number grew considerably. Nineteen FMRFamide-related peptides were identified in *H. americanus* 53, 25 FMRFamide-like peptides were identified in *C. maenas* using a combination of ESI-Q-TOF and direct tissue/off-line HPLC fractionation coupled with MALDI-FTMS analysis 54, and 14 FaLPs were identified in *Litopenaeus vannamei* through transcriptomic and mass spectrometry analysis 55. The authors identified most of the FaLPs in the VNC using a combination of two mass spectrometry approaches, as well as in the brain and eyestalk ganglia, the latter including the sinus gland. These neuropeptides are typically between 7 and 10 amino acids in length, excluding *N-*terminal extended forms (Table 1). For clarity, it should be again mentioned that at that time, several RFamide families, including MS, NPF, sNPF, and SK, were still partially grouped together. Among the RFamide families, MS, sNPF, and NPF are usually single-copy peptides or occasionally contain two putative bioactive peptides. Two bioactive peptides are typically encoded by the SK gene, with the bioactive peptides presenting post-translational modifications such as sulfation (-SO_3_) and/or *N-*terminal blocked forms like pyroglutamate. The so-called FaLPs are the only multicopy precursors, usually expressing more than three bioactive neuropeptides. In decapods, likely the most studied group among Crustacea, the precursor organization is relatively conserved, similar to that of coleopterans. Moreover, following the aforementioned studies, neuropeptides in other species were subsequently described, mostly using *in silico* predictions, such as in *Penaeus monodon* 56. In decapods, the precursors contain *N-*terminally extended YLRFamide, FLRFamide, and FIRFamide. In other crustaceans, the number of mature neuropeptides decreased considerably. In the water flea *Daphnia pulex* (Cladocera), the *firfamide* gene encodes two FIRFamides, confirmed by mass spectrometry 57. Similarly, in the copepods *Calanus finmarchicus* and *Tigriopus californicus*, one transcript was predicted *in silico* to encode two FIRFamides, and two transcripts were predicted to encode four putative mature FaLPs (three FLRFamides and one FVRFamide), respectively 58, 59. In the former species, *C. finmarchicus*, an FMRF receptor sequence was also predicted using homology searches with known receptors (see below for more details). In amphipods, a FaLP gene was predicted to encode six peptides in *Echinogammarus veneris* (three FL/IRFamides), as well as a second transcript that encodes eight peptides, including an RYamide-like peptide (NNRNLLRYamide; see 60 for details). This latter RYamide peptide is also present in several other amphipod FaLP precursors, such as *Gammarus chevreuxi* (comp62867_c0_seq1), suggesting a common feature in amphipods and ruling out errors in transcriptome assembly. Therefore, the FaLP precursors in crustaceans, like those in insects, contain a variable number of putative bioactive peptides, ranging from 2-4 in Cladocera and Copepoda to as many as 9 in decapods 53-55. According to functional experiments conducted in *D. melanogaster* 61, all the above-mentioned peptides should be capable of activating the FaLPs receptor. In *D. melanogaster*, it was shown that out of the eight possible putative bioactive peptides, all but one (FVRSamide) activate the receptor, including the one with an Arginine-to-Histidine replacement (FMHFamide). More recently, similar to what has already been described in insects, neuropeptide identification and distribution in decapods have also been investigated using mass spectrometry imaging (MSI). The cardiac ganglion of *C. borealis* was analyzed using a combination of MALDI-TOF-TOF imaging and Orbitrap 62. In addition to identifying a large number of peptides from several different neuropeptide families, including FaLPs, the study revealed the complexity of the cardiac ganglion's modulatory circuits. The authors suggest that future studies should combine the precise identification of neuropeptides via MSI with more detailed immunohistochemical resolution and localization to elucidate the specific functions of neuropeptides in the studied organ, as several aspects remain largely unknown. When comparing MSI with IHC, the main limitation associated with MSI is the fine-scale resolution, particularly in samples of relatively small size, as is common in arthropods. Currently, the primary limitation to achieving higher resolution is related to the spraying conditions: matrix crystals of about 20 μm prevent the use of smaller laser spot sizes (e.g., 15 μm). Although smaller laser spot sizes (5-10 μm) have been tested, they failed to generate a complete peptidome 19. On the other hand, MSI allows for the study of the co-occurrence of different neuropeptide families (including all para-copies of multicopy neuropeptides like FaLPs) and, through spatial segmentation analysis, the identification of tissues or compartments within organs associated with specific neuropeptide families. This capability, along with the reproducibility of MSI, could expand our current knowledge of the biological activity and functions of several neuropeptide families. Finally, despite the great accuracy in identifying immunoreactive cells and tissues using IHC, unless this approach is coupled with mass spectrometry, it is extremely difficult to distinguish between RFamide peptides in tissues where more than one neuropeptide family co-occurs.

### FaLPs in Chelicerata and Myriapoda

Among other arthropods, considerably fewer studies have been conducted on chelicerates. In this group, most research has focused solely on *in silico* predictions of both neuropeptide precursors and receptors. It is important to note that ligands and receptors are thought to co-evolve. However, in many of these groups, the ligands appear to have undergone greater variability compared to the receptors (see next section). In *Limulus polyphemus*, only a few genuine FaLP neuropeptides with FIRFamide have been identified, while the majority of other peptides have a MIRFamide *C*-terminus or others such as TIRFamide (Figure S1). Among mites, such as *Ixodes scapularis*, the FaLP *N-*terminal sequences appear to have evolved into YLHFamide, ILHFamide, IMHFamide, and MLHFamide (Figure S1), with a replacement of the arginine residues with histidine, as previously reported in *D. melanogaster* 61. In the groups Scorpiones and Araneae (Arachnopulmonata), most of the information has been obtained using *in silico* prediction. In Arachnida, the western black widow spider *Latrodectus hesperus* is used as a model to search for neuropeptides in other species 2, 63. In *L. hesperus*, more than 20 FaLP peptides with *C*-termini IMRFamide, MMYFamide, and MIHFamide were identified. In Scorpiones, *in silico* predicted peptides with a *C*-terminus consensus sequence similar to other chelicerates were identified. The FMRFamide gene of *Centruroides sculpturatus* encodes several peptides with *C*-termini IMRFamide, LIRFamide, MIHFamide, LMHFamide, LIHFamide, and IMHFamide (Figure S1). Finally, in Myriapoda, the predicted FaLP precursor gene of* Hanseniella nivea i*s similar to that of Pancrustacea, encoding several FLRFamide peptides (Figure S1). Most of the above-mentioned peptides need to be corroborated by both mass spectrometry analyses and functional studies in the future.

## Receptor structure

### FaLP receptor in insects

**Figure 2:**
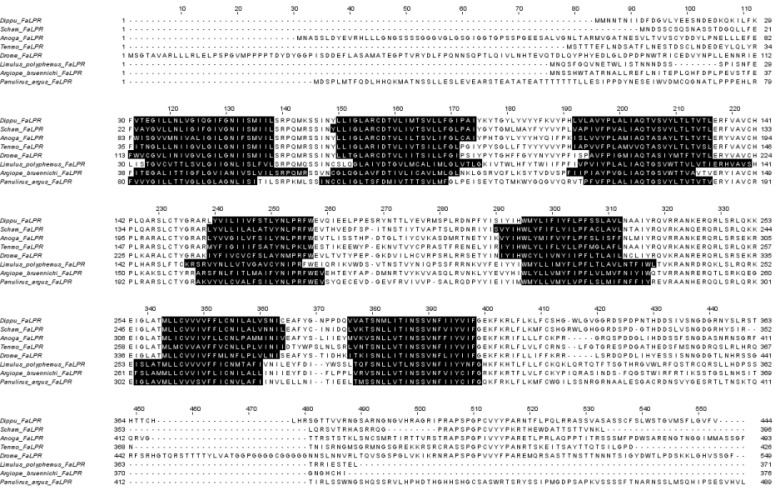
Alignment of five insect, two chelicerata and one crustacean FMRFa-like peptide receptors from *Diploptera punctata* (DippuFaLPsR, KAJ9593213.1), *Schostocerca americana* (SchamFaLPsR, XP_046985417.1), *Anopheles gambiae* (AngeFaLPsR, AAQ73620.1), *Tenebrio molitor* (TenmoFaLPsR, KAJ3627863.1), *Drosophila melanogaster* (DromeFaLPsR, NP_647758.1), *Limulus polyphemus* (XP_022239599.1), *Argiope bruennichi* (XP_055950503.1) and *Panulirus argus* (Christie 2020). The analysis was conducted using the DeepTMHMM server 93, accessed in January 2025. Colour black indicates seven transmembrane helices.

The signalling pathways of FaLPs have been studied in a several species, including *A. gambiae* 5, *R. prolixus* 4, *Baculum extradentatum* 6 and *D. melanogaster* 64. The FaLP receptor (FaLPR) in insects was initially discovered and characterized in *D. melanogaster* in 2002 61, 65. This discovery paved the way for further characterization and structure-activity studies in the fly over the next decade 66, 67. Like other insect neuropeptide receptors, FaLPRs belong to GPCRs composed of seven transmembrane (TM) helixes (Figure 2). These helices are connected by six loops, three of which are extracellular while three intracellular interact with the G protein. The structure includes also an *N*-terminal extracellular segment and an intracellular *C*-terminus. Ligands can attach to transmembrane helix binding sites or to extracellular loops and *N*-termini 68. In insects, neuropeptides act through GPCRs 69 followed by the generation of soluble second messengers such as cyclic adenosine monophosphate (cAMP) 70-72 or inositol 1,4,5-trisphosphate (IP_3_) 73, 74. IP_3_ binds to and activates the endoplasmic reticulum (ER)-localized Ca^2+^ channel, the IP_3_ receptor (IP_3_R), resulting in the release of calcium from ER stores 75. FaLPRs, among others, are involved in this signalling cascade 73, 74, 76. In other arthropods the FaLPs act by the same mechanism, activating their GPCRs. The analyses conducted by Rasmussen *et al.* 77 showed that FaLPRs belong to subfamily A of the GPCRs rhodopsin-like family in which ligands bind to the binding site within transmembrane helices. Thus, the receptors for FaLPs contain typical rhodopsin-like amino acid patterns, e.g., GN in helix 1, LX3Din helix 2, D(E)RY(F) in helix 3, CNL(I)X2 in helix 6 and NFX2Y in helix 7, as shown in *T. molitor* and *Z. atratus* 78. These sequences comprise molecular switches that are responsible among others for propagation of a hydrogen-bond network from the ligand binding pocket to the intracellular side of the receptor upon activation, are essential in holding the receptor in an inactive state and preventing access to the G protein-binding site through interactions between TM3 and TM6 or are important in binding the agonist in order to induce structural changes near TM5 and TM6 which lead to receptor activation (for details see 77). However, in other invertebrates FaLPs can also act through other types of receptors. In mollusc, *Aplysia californica* it can directly bind and activate the ionotropic receptor for sodium (FMRFamide-activated sodium channels (FaNaCs))^79^. Although peptide-activated ion channels are rare, authors of another study from 2022, do not dismiss the possibility that other FaLP/FMRFa-gated channels can be found in other clades 80. However, this needs thorough and deep research. To date, using at least *in silico* methods, FaLPRs have been identified in most insects, including flies, mosquitoes, moths, kissing bugs, leaf bugs, and beetles 68, 77, 78, 81.

#### Receptor localisation

In most of the research, the distribution of FaLPRs was studied for the first time and the most in *D. melanogaster* 65, followed by mosquitos 5 and beetles 78. Meeusen *et al.* 65 demonstrated that Drome-FaLPsR is present in the nervous tissues, guts, ovaries, trachea, and fat body of fruit flies. This indicates that FaLPs signalling is involved in the regulation of numerous physiological processes. Studies on two beetles, *T. molitor* and *Z. atratus,* showed similar spatial distributions to *D. melanogaster*, revealing FaLPRs in the central nervous system (brain, ventral nerve cord (VNC), RCC), heart, hindgut, and reproductive organs of both males and females (ejaculatory duct and oviduct) 78. Together with the fact that the application of synthetic peptides affects the contractile activity of the above-mentioned organ muscles, it can be concluded that signalling through FaLPRs modulates the activity of these organs. On the other hand, in *A. gambiae* in the abdomen, the expression of FaLPR was very low, whereas samples from the head and thorax of this insect showed high receptor abundance 5. The fact that in mosquitos, the receptor in the abdomen is present in low quantities is unusual since, FaLPs immunoreactivity was observed in the abdominal ganglia, midgut, and other visceral organs as well as in abdominal neural processes in *D. melanogaster*, *R. prolixus*, *S. gregaria*, *Phormia regina* and other insects 4, 82, 83, suggesting that the FaLPRs are also highly expressed in the abdomen of *A. gambiae*. This might be due to the specific way that mosquitoes digest food in comparison to beetles or flies.

### FaLP receptor in crustaceans and chelicerates

FaLP receptors in crustaceans exhibit a widespread distribution throughout their nervous systems, including the brain, ventral nerve cord, and stomatogastric nervous system 58, 84. It should be noted, however, that in the case of studies on *Calanus finmarchicus*, although the authors claim to have identified a true member of the FaLP receptor family, they were only able to predict five transmembrane domains (whereas GPCRs are typically characterized by 7 TMs) 58. Additionally, in certain crustaceans, such as *Panulirus argus*, there are two isoforms of the FaLPs receptor. Unlike other receptor groups where diversity arises from multiple genes, the diversity of FaLP receptors appears to result from alternative splicing of a single gene 84. Consequently, more detailed studies are required to precisely characterize these receptors in crustaceans.

In Chelicerata studies focusing on the identification of FaRP receptors are scarce. However, the distribution of FaLPs themselves seems to be similar as in crustaceans. For example, studies on the tropical wandering spider *Cupiennius salei* have shown that FaLPs are expressed in all ganglia of the CNS, with varying expression levels among different neurons 85. The presence of FaLPs throughout the nervous system implies that their receptors should be similarly distributed to mediate the neuromodulatory effects of these peptides. However, further research is necessary to map the precise localization of FaLPRs and fully understand their functional roles.

In general, when available databases in NCBI were searched for FaLPR, they have been found in different arthropods groups (Supplementary material 1). Phylogenetic analysis showed similarities between the receptors. The phylogenetic tree constructed using different FaLPs receptors is in agreement with recent arthropod phylogeny, supporting the idea that receptors co-evolved with species while maintaining physiological functions (Supplementary material 1). Among arthropods, the only group in which it was not possible to identify a FaLPR was the Parasitiformes (e.g., *Ixodes*). Since a precursor sequence for the FaLPs was identified in this group, it is evolutionarily unlikely that the receptor was lost. In this case, one might be tempted to suggest that the receptor was lost as a consequence of the parasitic lifestyle associated with mites. However, this hypothesis would be unlikely, especially for a neuropeptide family that is widely distributed. Therefore, it is more likely that the receptor was not sequenced, or that the receptor sequence is highly derived and cannot be identified using a homology search strategy. Alternatively, it is also possible that the FaLPs neuropeptide of the Parasitiformes can activate other RFamide receptors, but this needs further investigation.

## Physiological properties of FaLPs in insects

### Heart and skeletal muscle contractions

**Figure 3:**
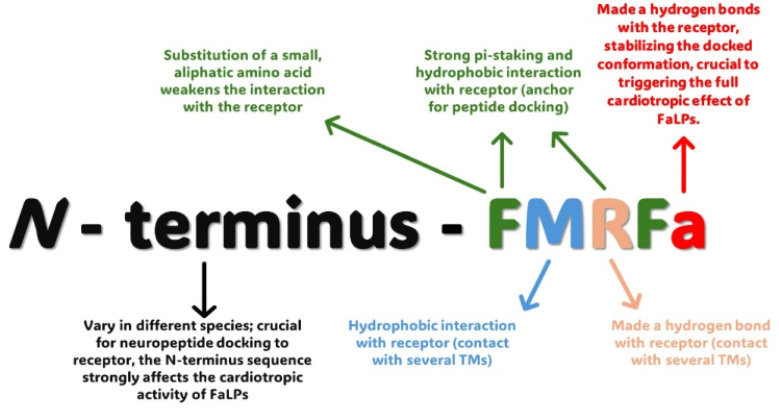
Summary of the interactions between insects FaLPs with FaLPs receptor based on the *in silico* analysis performed by Maynard *et al.* 66.

One of the well-known physiological properties of FaLPs in insects is their myotropic activity on the insect heart and visceral/skeletal muscles 9, 94. Generally, direct and indirect regulation of muscle contractions seems to be a major physiological activity of FaLPs 78. Notably, the first documented property of these neuropeptides was the cardioacceleratory action of the FMRFamide peptide on the heart of *M. nimbosa* 1. Further research carried out on insects indicated that the cardioregulatory effects of extended FMRFamides may be dependent on the tested insect species, the amino acid sequence of the bioanalogue, and the applied concentrations 9, 78, 94. Research on this issue showed that the tested analogues of FMRFamides evoked a stimulatory or inhibitory effect but also a biphasic response characterized by an initial increase in heart rate, which results in a cardioinhibitory effect. For example, FMRFamide injection increased the heart rate of the mosquito larva *A. gambiae* 95. Additionally, Hillyer *et al.* 5 revealed that the injection of FMRFamide at a concentration of 10^-6^ M increased the heart rate of adult *A. gambiae*. However, the application of this peptide at a concentration of 10^-2^ M led to a significant decrease in the anterograde and retrograde heart rate in the mosquito 5. Interestingly, a high concentration of FMRFamide influences the proportion of anterograde and retrograde contractions by twofold increasing the percentage of retrograde contractions after FMRFamide injection at a concentration of 10^-2^ M 5. Similar results were observed after the application of SALDKNFMRFamide; however, the authors postulated that the cardioinhibitory effects are nonphysiological because they occur at concentrations much higher than those normally observed in insect haemolymph 5. A similar dose dependent, bimodal action of FaLPs was also observed in other insects, for example, in the locust *S. gregaria* research by Cuthbert & Evans 96 showed that the application of one of the tested FaLP analogues (YGGFMRFamide) at high concentrations (above 10^-7^ M) caused an immediate increase in the heart rate of *S. gregaria*. After this period, the heart rate of the locust decreased. A cardioinhibitory effect was observed until YGGFMRFamide was removed from the superfusate 96. Interestingly, at lower concentrations (up to 10^-7^ M), the cardioaccelerator effect of FaLPs was observed in *S. gregaria* only in the case of hearts characterized by regular beating 96 The cardioacceleratory property of FaLPs was also observed in beetles. Research by Marciniak *et al.* 78 showed that the application of FMRF6 (NSNFLRFamide) led to a dose-dependent increase in the heartbeat frequency of the semi-isolated heart of *Z. atratus*. In contrast to that of *Z. atratus*, the heart of *T. molitor* reacts differently, and the application of FMRF6 elicits an immediate cardioinhibitory effect, but statistically significant changes in heart rate were observed only at higher concentrations (10^-5^ M). Structure-activity studies have shown that *C*-terminal amidation seems to be crucial for the cardiostimulatory action of FMRFamides 97. Intriguingly, the amidation of the *N*-terminus increases the potency of FMRFamide-like peptides 96. Further results presented by Cuthbert & Evans 96 also support the supposition about the significance of the *N*-terminus structure for the cardioregulatory action of FaLPs. For example, the threshold value for the lobster FMRFamide extended analogue (TNRNFLRFamide) was 1000 times lower (10^-10^ M) than that for the locust FaLPs. Moreover, the effect elicited by lobster peptides on the locust heart was much stronger. Furthermore, the results obtained by Duve *et al.* indicate that even single amino acid replacement can be crucial for the cardioregulatory properties of FaLPs. For example, Calvi-FMRF6 (ASGQDFMRFamide) compared to Calvi-FMRF5 (APGQDFMRFamide), is not active. This result suggested that the substitution of serine with proline at the second position is crucial for the cardioacceleratory activity of FaLPs 97. Authors proposed that these differences in the physiological activity of FaLPs could also be related to their activation of different receptors by those neuropeptides. This supposition was partially confirmed by Johnson *et al.* 98, who showed that DPKQDFMRFamide elicits effects that are mediated by both the FaLPs receptor and a myosuppressin receptor. These results can explain why cardioacceleratory effects are usually not very strong and are sometimes missing. *In silico* research by Maynard *et al.* study in detailed docking of the FMRFa and other FMRFa-like peptides to the FMRF receptor and their cardiotropic activity on *D. melanogaster* heart 66. The authors mostly focused on the importance of *C*-terminus and *N*-terminus modifications during interaction of FaLPs with their receptor (Figure 3) 66. However, further research is needed to fully evaluate the mode of action of FaLPs on the heart of this insect. It should also be mentioned that the close connection between FaLPs signalling and the regulation of heart physiology was also confirmed by the results of immunohistochemical studies showing the localization of nerves exhibiting FMRFamide-like immunoreactivity. For example, research by Calvin and Lange 99 on the stick insect *Baculum extradentatum* revealed FMRFamide-like immunoreactive staining *inter alia* in nerve branches of segmental nerves projecting to the heart and nerves innervating the heart ostia 99. For example, research by Calvin and Lange 99 on the stick insect *Baculum extradentatum* revealed FMRFamide-like immunoreactive staining *inter alia* in nerve branches of segmental nerves projecting to the heart and nerves innervating the heart ostia 99.

FMRFamide-like peptides are not only important for the regulation of heart contractility. Investigations concerning the myotropic activity of these neuropeptides indicate that FaLPs are crucial for the regulation of motoneuron activity and skeletal muscles. For example, research by Evans & Myers 100 conducted on the locust *S. gregaria* showed that FMRFamide and related peptides modulate the twitch tension of the extensor-tibiae muscle by stimulating the slow excitatory motor neuron (SETi). Furthermore, the effect of FMRFamide on the tension induced by SETi was additive to the stimulatory effect elicited by octopamine and proctolin 100. Interestingly, FMRFamide did not affect the tension induced by the fast motor neuron (FETi) 100. Further research on this issue showed that the stimulatory effect of FMRFamide on slow muscle fibres, similar to its effect on heart contractility, is dose dependent 100, 101. Additionally, similar observations were reported for FMRFamides identified in *D. melanogaster*. Research by Hewes *et al.* 102 showed that different FMRFamide bioanalogues processed from FaLPs precursor significantly affect the twitch tension of *Drosophila* larval body wall muscles 102. Intriguingly, for the mixtures of tested FMRFamide analogues, the muscle response was stronger than that for the FMRFamide analogue alone 102. Additionally, one of the tested analogues, DPKQDFMRFamide, increased the amplitude of the excitatory junctional current (EJP), which may suggest that FaLPs improve synaptic transmission 102. The effect of DPKQDFMRFamide on *Drosophila* muscle was also confirmed, for example, by Dunn and Mercier 103, who showed that DPKQDFMRFamide selectively modulates the amplitude of the EJP associated with motor neurons innervating the ventral longitudinal muscles of *D. melanogaster*. The effect of FaLPs on insect muscle contractions was also confirmed at the organismal level. Research by Klose *et al.* 73 proved that the application of DPKQDFMRFamide enhances the fictive locomotion of *Drosophila* flies. On the other hand, the FMRFamide analogues F(D-MET)RFamide and YGGFMRFamide did not significantly affect the neurally evoked contractions or resting tension of the coxal depressor muscles of the cockroach *P. americana* 104.

The structure-activity studies performed, for example, by Walther *et al.* 105 and Evans & Myers 100, showed that the *C*-terminal sequence is essential for the activity of skeletal muscles. Walther *et al.* 105 showed that FMRFamide and YGGFMRFamide activity is maintained when methionine is replaced by another neutral amino acid but not when it is replaced by an acidic one. Additionally, research by Cuthbert and Evans 96 showed that FLRFamide and FMRYamide can induce submaximal effects at concentrations of 10^-5^ M. Furthermore, FPRFamide, RMRFamide, FGRFamide, and pQGRFamide (anthoRFamide, an anthozoan peptide) did not affect the twitch tension of the extensor tibiae muscle in the locust *S. gregaria*. In addition, FMRF analogues without *C*-terminal amidation did not have stimulatory effects on locust muscles too 96. Research by Evans and Myers 100 revealed that the *C*-terminal dipeptide RFamide is also not active. These authors also noted the important role of the *N*-terminal sequence in shaping muscle activity via FaLPs. Similar to research conducted on insect hearts, the extended form of YGGFMRFamide is much more potent than FMRFamide 96, 100.

The role of FaLPs in the functioning of skeletal muscles was also confirmed by immunohistochemical staining. Several investigations have shown that antibodies against FaLPs or RFamides bind to neurons that innervate insect skeletal muscles 104. Furthermore, research conducted on the cockroach *P. americana* revealed numerous stained cell bodies in the methathoracic ganglion and peripheral nerves that contain motor and sensory neurones. Axons of these neurones, for example the femur-tibia, joint in *P. americana*. Additionally, Myers and Evans 82 found FMRF-like immunoreactive (using bovine pancreatic polypeptide (BPP) antiserum) neurons associated with muscles in the locust *S. gregaria*. Authors have shown that immunoreactive nerves project to spiracle muscles or dorsal longitudinal flight muscles 82.

Despite wide knowledge concerning the role of FaLPs in the activity of insect muscle, the strict mode of the regulatory action of these neuropeptides has not been fully characterized. Recent research has shown that FaLPs act via GPCR receptors 67. However, as we mentioned previously, FMRFamide may activate not only specific FaLPs receptors but also receptors for myosuppressin 98. These results are supported by those of Klose *et al.* 73, who demonstrated that DPKQDFMRFamide enhanced synaptic transmission in *D. melanogaster* neuromuscular junctions through activation of the FaLP receptor and Dromyosuppressin Receptor-2 (DmsR-2). On the other hand, Milakovic *et al.* 67 showed that the frequency of muscle contractions in *Drosophila* larvae induced by DPKQDFMRFamide was significantly decreased in individuals with reduced expression of the gene encoding the FaLPs receptor. However, Milakovic *et al.* 67 did not suggest the involvement of myosuppressin receptors in the induction of myotropic effects after the application of DPKQDFMRFamide.

After activation of the FaLPs receptor, the myotropic activity of FaLPs is closely associated with Ca^2+^ influx. Research by Clark *et al.* 106 showed that the application of nifedipine and nicardipine, which are potent blockers of L-type channels, completely abolished the effect of DPKQDFMRFamide on *Drosophila* muscles. However, T-type channel blockers did not cause a similar effect. The close connection between the Ca^2+^ and FaLP modes of action was also postulated by Klose *et al.* 73, who revealed that the application of DPKQDFMRFamide increased the presynaptic Ca^2+^ response and the quantal content of the released transmitter.

Moreover, Dunn and Mercier 103 showed that calmodulin-dependent protein kinase II (CamKII) is an important effector protein in FaLPs signalling. However, Milakovic *et al.* obtained different results and negated the role of CamKII in the myomodulatory action of FaLP 67. Interestingly, in both studies, the authors used the same model organism (*D. melanogaster*, larvae from the third stage of the wandering stage) and used a similar method of confirmation of CamKII involvement in FaLP signalling, including analysis of the effect of the same neuropeptide (DPKQDFMRFamide) and CamKII inhibitor (KN-93). The only significant difference is the fact that Dunn and Mercier 103 tested presynaptic effects by evoked EPJ recorded in the ventral longitudinal muscles, but Milakovic *et al.* 67 focused on the postsynaptic effect and analysis of muscle tonus and contractions. As Milakovic *et al.* suggested the postsynaptic and presynaptic effects of a modulatory substance might be regulated by different intracellular signalling pathways and receptors. Furthermore, based on the literature and their own research, authors postulated a possible model of DPKQDFMRFamide activity based on the fact that this peptide activates the G protein, whose subunits act directly on L-type channels to stimulate Ca^2+^ influx. Ca^2+^ influx subsequently elicits skeletal muscle contraction via the calcium-induced release of calcium from the sarcoplasmic reticulum 67. The involvement of other second messengers, such as cyclic nucleotides, IP3, arachidonic acid, and phospholipase C (PLC), was negated in research conducted on *S. gregaria* and *D. melanogaster* 67, 100, 101. However, the effects of some second messengers, such as linoleic acid, have still not been tested.

### Circadian clock regulation

FaLPs have been implicated in the regulation of circadian rhythms in insects. Soechler *et al.* 107 revealed the presence of FaLPs in the accessory medulla (AMe), which is known as the circadian clock that controls circadian locomotor activity rhythms in *Leucophea maderae*. FMRFamide-like immunoreactivity was found in four of the six soma groups associated with the AMe, as well as in most neuropils of the protocerebrum. In the same study, the injection of Peram-FMRFa-7 (DRSDNFIRFamide), previously identified in *P. americana* 44, caused time-dependent phase changes in the locomotor activity rhythms of *L. maderae* cockroaches at circadian times 4 and 8 (CT 4 and CT 8). The change caused by Peram-FMRFa-7 at CT 4 was also positively correlated with the dose of this peptide, as phase delays decreased with decreasing amounts of the peptide injected. These results suggest that this peptide could be responsible for activity changes during the early day 107.

It was also shown that FaLPs and the FaLPR are involved in modulating the sleep induced by cellular stress. *Drosophila* mutants for FaLPs or its receptors were shown to have a reduced recovery sleep caused by heat stress or infection with *Serratia marcescens* and became infected more quickly than controls 108.

FaLPs are also supposed to play an important role in insect diapause. Hao *et al.* 109 have detected FaLPs encoded by the *Flrf* gene under a short and long photoperiod in the CNS of *L. migratoria*. The presence of two of them (GSERNFLRFa and DRNFIRFa) was detected only in the group of insects treated with a short photoperiod, suggesting their role in the induction of photoperiodic diapause. Their results showed that the *Flrf* gene promotes diapause in locusts 109. They also proposed that FaLPs expressed from the *Flrf* gene are regulated by the photoperiod and subsequently activate downstream FOXO and ROS to induce diapause of *L. migratoria*, but specific mechanisms of this regulation are still unknown.

### Reproduction

FaLPs activity also occurs in the insect reproductive system. In the case of male insects, transcripts for extended FMFRamide receptors have been identified in various parts of this system in *R. prolixus*, with the highest expression in the *vas deferens* and the ejaculatory duct 110. The same authors also reported FMRFamide-like immunoreactivity in other reproductive organs, such as seminal vesicles and accessory gland ducts, of this insect. The presence of the receptor for extended FMRFamide was also confirmed in the ejaculatory ducts of two beetle species, *T. molitor* and *Z. atratus* 78.

Further *in vitro* tests on the ejaculatory duct, transparent accessory gland, and seminal vesicle muscles confirmed these results, showing a concentration relationship between the activity of AKDNFIRFamide and an increase in contractile activity, expressed as an increase in the frequency of contractions 110. Marciniak's research in which NSNFLRFamide was applied to the muscles of the ejaculatory duct of the beetle *T. molitor* showed a high (over 70%) but irregular increase in the frequency of contractions 78.

In females, FaLPs immunoreactivity (FLI) have been detected in the calyx of kissing bugs, but their presence in the ovaries and ovarioles has not been confirmed 4. However, processes related to FMRF-like immunoreactivity were observed in the oviduct, both lateral and common parts, *bursa copulatrix*, and *spermatheca* 4. Similar effects were observed in *L. migratoria,* in which FLI was observed in the muscles of *spermatheca* 111. This was partially confirmed in *T. molitor* and *Z. atratus,* in which the presence of the receptor for extended FMRFamide was confirmed 78.

Physiological bioassays confirmed the described results. The application of AKDNFIRFamide and GNDNFMRFamide to organs such as ovaries and oviducts caused a dose-dependent increase in muscle tone 4, similar to what was observed in the case of *L. migratoria* and YGGFMRFamide, which led to an increase in the contraction of oviduct muscles 111. The application of NSNFLRFamide on the oviduct of the beetle *T. molitor* caused an increase in the frequency of muscle contractions 78. After the application of FaLPs to organs such as the ovaries, the presence of receptor transcripts for these neuropeptides has not been demonstrated 4.

### Other physiological properties

FaLPs are also involved in energy balance regulation and digestion and can alter the activity of digestive enzymes. Hill and Orchard 112 revealed that endogenous FaLPs, GQERNFLRFamide and AFIRFamide, similar to locusta acronym MS (previously designated as SchistoFLRFamide), increased total amylase and α-glucosidase activity *in vitro* in the midgut lumen of *L. migratoria*. However, GQERNFLRFamide and AFIRFamide did not cause significant changes in the total activity of digestive enzymes in tissue extracts at the tested concentration of 10^-8^ M 112. In a previous study, GQERNFLRFamide was shown to have no effect on midgut muscle contractions 113. It has also been shown that FMRFamide and its cognate receptor FMRFR mediate the fat loss effect of dietary cysteine in *D. melanogaster*. When cysteine consumption is increased, FaLP triggers lipid degradation by activating FaLPR and the downstream PKA pathway in fat body cells, while at the same time, in the CNS of flies, the peptide and receptor reduce the sensitivity of sweet-sensing gustatory neurons and hence food consumption 114. FaLPRs have also been associated with the larval to pupal transition under nutrient-limiting conditions 74.

Other investigations have shown that FaLPRs are also responsible for the modulation of insect flight. *D. melanogaster* mutants of the gene encoding Drome-FMRFR exhibit significant flight deficits associated with dopaminergic cells. The downregulation of expression of gene for the receptor by specific RNAi in adult central dopaminergic neurons results in the progressive loss of sustained flight 76. In addition to the regulation of visceral muscle activity, FaLPs and their receptors have been associated with an escape response to intense light in *Drosophila* larvae 73. Exposure to bright light is a stress factor, and a quick reaction protects larvae from desiccation. The receptor, as well as a receptor for dromyosuppressin, is required for this behaviour, as the knockdown of these receptors causes a significant reduction in locomotor activity during the escape response 73. Knockdown of the FaLPs receptor in *D. melanogaster* causes a reduction in heat stress- or infection-induced sleep and reduces startle-induced locomotor activity 108, 115, 116.

## Physiological properties of FaLPs in crustaceans

As mentioned above FaLPs in crustaceans have been isolated from diverse neurosecretory and nervous tissues, such as pericardial organs, eyestalks, the brain, and the thoracic, stomatogastric and commissural ganglia 48, 49, 51-54, 117-127.

The physiological activity of the crustacean FaLPs have been studied in different species, including the lobster *H. americanus*, the crab *C. sapidus*, *C. magister*, *Cancer productus*, *C. borealis*, *Carcinus means*, the crayfish *P. clarkii*, the giant freshwater prawn *M. rosenbergii*, and the giant tiger prawn *P. monodon* (Table 2). FaLPs have been shown to regulate many systems in these animals, including circulatory system, stomatogastric nervous system, and muscle system 12, 128, 129.

### Circulatory system: influence of FaLPs on the heart

The first FaLPs identified in crustacean, lobster *H. americanus* pericardial organs were TNRNFLRFa (F1) and SDRNFLRFa (F2) 49. These peptides were tested on *C. means* heart and have positive chronotropic and negative inotropic effects with reduced stroke volume, arterial pulse pressure and ventricular pressure 130. Mercier and Russens 131 used an isolated heart from *P. clarkii* to test TNRNFLRFa and SDRNFLRFa. An increase in the rate and amplitude of heartbeats was observed. McGaw *et al.* 132 and McMahon 133 tested SDRNFLRFa and TNRNFLRFa *in vitro* and *in vivo* on the *C. magister* heart. Both peptides caused cardioexcitation when they were semi-isolated and, in contrast, had cardioinhibitory effects *in vivo*. The TNRNFLRFa peptide was also tested in *H. americanus* hearts and was shown to increase the rate and strength of the heartbeat 134. The SDRNFLRFa peptide tested on *H. americanus* ostial valve muscle caused an increase in the amplitude of electrically evoked contractions 135. Similar to *H. americanus* peptides, two *P. clarkii* peptides (DRNFLRFa - DF2 and NRNFLRFa - NF1) were found to increase the heart rate and amplitude of the semi-isolated *P. clarkii* heart 51, 136. Krajniak 48 isolated and identified GYNRSFLRFa from crab *C. sapidus*. The peptide caused a dose-dependent increase (10^-9^-10^-5^ M) in crab heart rate. Fort *et al.* 137 examined the effects of TNRNFLRFa, SDRNFLRFa, and GYNRSFLRFa on *C. sapidus* heart. All the tested FaLPs showed both chronotropic and inotropic positive effects. In 2006, Cruz-Bermudez *et al.* identified and sequenced the neuropeptide GAHKNYLRF from *C. borealis, C. productus,* and *C. magister* 121. This peptide increased the frequency of bursts and the number of spikes per burst of the isolated cardiac ganglion in *C. borealis*. When one sums up the effects of different FaLPs on the crustacean's circulatory system it is rather clear that these peptides pose cardioaccelerator activity (Table 2) which is similar to insects (Table 3). To date any tests were done whether these peptides are influenced in regulation of metabolism. However, regulation of heart activity highly likely indirectly influence other physiological processes in crustaceans.

### Stomatogastric nervous system: influence of FaLPs

Skiebe 138 reviewed the peptidergic modulation of the stomatogastric nervous system in crustaceans. Thus, this review only briefly describes the functions of FaLPs in the stomatogastric nervous system. In 1993, Weimann and Marder isolated and sequenced two FaLPs, TNRNFLRFa and SDRNFLRFa, from extracts of the stomatogastric nervous system of the crab *C. borealis*, which were previously found in the pericardial organs of *H. americanus* 50. Both peptides activated gastric motor patterns in the crab. Pyloric and gastric mill activation was also observed in several other studies 50, 139-142. As with the circulatory system FaLPs cause a stimulatory effect on gastrointenstinal tract in crustaceans. However, it is not known whether they activate the receptor, or this is somehow indirect effects. Increased activity of digestive tract can be related to increased activity of circulatory system what in turn may help in absorption and distribution of nutrients.

### Muscle system: influence of FaLPs

FaLPs modulate somatic muscle contractions and neuromuscular transmission in crustaceans in the same manner as they do in insects (Table 3). Mercier *et al.* 143 tested TNRNFLRFa and SDRNFLRFa in the phasic extensor muscles of the abdomen of *P. clarkii* and reported an increase in nerve-evoked tension and excitatory postsynaptic potentials. Meyrand and Marder 144 tested FaLPs on the pyloric dilator muscle of the shrimp *Palaemon serratus* and observed sequences of rhythmic depolarizations and contractions. Worden *et al.* 134 again examined the TNRNFLRFa in nerve-muscle preparations from *H. americanus.* This peptide induced tonic contractions of the exoskeletal muscle in the absence of nerve activity, which suggests direct action on the dactyl opener muscle. Skerrett *et al.* 136 examined NRNFLRFa and DRNFLRFa in the neuromuscular junctions of the deep abdominal extensor muscles of *P. clarkii.* The two peptides increased the amplitude of excitatory junctional potentials in the muscles tested.

## What is really known about physiological properties of FaLPs in other arthropods?

As was already described, the number of reports regarding the identification of FaLPs transcripts in tissues of various Arthropod species, other than insects or crustaceans, is increasing 2, 85, 117, 145-147. However, information about the physiological properties of these peptides has been still very limited.

### Ticks

FaLPs were shown, using the immunocytochemical method, to be broadly distributed in the synganglion of two tick species *Ornithodoros parkeri* and *Dermacentor varisbilis* 148. The results of the analysis of synganglion transcriptomes of other tick species (*Ixodes scapularis, Rhipicephalus microplus*) seem to confirm these findings 146, 149. It was also revealed that FaLPs were present in neurons that innervate the coxal muscles in *O. parkeri*, suggesting their involvement in locomotor control 148 what was also proved in other arthropods.

### Spiders

Using the polyclonal antibody against FMRFamide-like peptides, wide distribution of FaLPs were observed in the central nervous system, especially in the visual neuropils of the spider *Cupiennius salei* 147, 150. They were also found in mechanosensory neurons and nerve fibres projecting to each leg ganglion in *C. salei* 147. Fabien-Fine and co-workers showed that in some central and mechanosensory neurons of *C. salei* FaLPs co-loaclizedlocalized with acetylcholine 147. Furthermore, groups of neurons in which FaLPs co-localized with glutamate were observed in this species 85. This suggests that these molecules can be co-released and FaLPs could be engaged in synaptic transmission as a neurotransmitter and/or a neuromodulator.

### Horseshoe crab

FaLPs immunoreactivity has been found in the central and peripheral nervous system and muscles of the horseshoe crab *Limulus polyphemus* 151-153. The extensive distribution of these peptides in nervous tissue indicates a possible role of these peptides in synaptic transmission or modulation of pathways in the CNS.

The crustacean FaLPs (TNRNFLRFamide; SDRNFLRFamide, GYNRSFLRFamide) were tested on the heart of *L. polyphemus* and caused chronotropic and inotropic stimulation of the heartbeat in a dose-dependent manner. This was also show in crustaceans (see above). They also increased the excitability and contractility of the cardiac muscle fibers. TNRNFLRFamide and SDRNFLRFamide enhanced excitation of cardiac ganglion neurons by increasing the burst rate 154. FaLPs fractions isolated from the brain of *L. polyphemus* also caused an increase in the frequency of the heart rate as well as the amplitude of contractions of the heart of the horseshoe crab 151, 154. The results of another study suggest that FaLPs can be released by the intestinal nerve axons and regulate the motility of the gut of *L. polyphemus*. The crustacean FaLPs relaxed the gut of *L. polyphemus*, and also decreased its contraction activity induced by proctolin. The effect of the intestine relaxation was also obtained by applying FaLPs fractions from HPLC on the *L. polyphemus* midgut preparations 153.

## Similarities between insects FaLPs and mamallian peptides from -RF family

In mammals, five genes encode RFamide peptides. These peptides are expressed in the brain and some peripheral tissues 155. The first two mammalian RFamide-like peptides, the neuropeptides FF and AF, have been isolated from the bovine brain 156. Currently, the increasing number of RFamide peptides found in mammals can be subdivided into five groups: the neuropeptide FF (NPFF) group, RF-amide related peptide (RFRP) group, pyroglutamylated RF-amide peptide (QRFP) group, kisspeptin/metastin group and prolactin-releasing peptide (PrRP) group 157.

RFamide peptides, similarly as FaLPs in insects, are widely distributed within the central and peripheral nervous system. Moreover, they have been detected in many peripheral tissues and organs, such as the spleen, lungs, heart, pancreas, adrenal glands, placenta, pituitary gland, thymus, and macrophages 155, 157-163. The distribution of members of different RF-amide peptide groups varies and shows species specificity.

The activity of RFamide peptides results from the ability to activate a wide group of RFamide peptide receptors. Several of these proteins have been identified, including NPFFR1, NPFFR2, QRFPR, prolactin-releasing peptide receptor (PrRPR), and Kiss1R. All of these receptors are GPCRs and commonly activate G_i/o_ signal transduction pathways 159. The selectivity of receptors for binding a specific ligand is quite high, and RFRP activates NPFFR1, NPFF interacts with NPFFR2, QRFP interacts with QRFPR, PrRP interacts with PrRPR, and kisspeptin binds to Kiss1R. Nevertheless, some RFamide peptides can activate more than one type of receptor, where NPFFR1 and NPFFR2 can be activated by all RF peptides but with various efficiencies, whereas QRFPR, PrRPR, and Kiss1R cannot, and their affinity for endogenous peptide ligands is very strict 158.

The wide distribution of RFamide peptides and their receptors within the nervous system and peripheral tissue confirms their important role in the regulation of many physiological processes. This is also the case for FaLPs. RFa peptides play an equally important role in the central processing of visceral autonomic signals related to feeding, energy balance, generation of central cardiovascular responses, muscle contraction, reproduction, nociception, neuroendocrine regulation, response to stresses, and behaviour 158, 161. Such wide physiological activity is like that of insects and other arthropods, especially in terms of regulation of muscle contraction activity, feeding and reproduction (Figure 3). Further studies revealing not only physiological but also structural similarities on the level of precursors and receptors between FaLPs and mammalian RFamides are needed. On the other hand, the current knowledge about the physiological functions of FaLPs is discontinuous. Different cell specific processing of the precursor has not been shown thus further studies about the role of different bioanalogues in the precursor is of interest, especially when only one receptor is present.

As neuropeptides are mainly considered as activators of G-protein coupled receptors, the FMRFa is a notable example of a neuropeptide which can directly activate also a ionotropic receptors 79, 80. An example are FMRFa-gated sodium channels (FaNaC). They belong to the epithelial sodium channel/degenerin (ENaC/DEG) superfamily of cation channels 79, 80. This superfamily includes seven families, three of which were first described in vertebrates: ENaC, Acid-Sensing Ion Channels (ASICs), and Brain-Liver-Intestine Sodium Channel (BLINaC)/Human Intestine Sodium Channel (hINaC). Other ENaC/DEG families were reported in invertebrates: the Degenerins from *C. elegans*, the *Drosophila* PPK channels, the FMRFamide-gated Sodium Channel (FaNaC) from mollusks and annelids, the FLR-1 receptor that was only identified in *C. elegans* and *Hydra*-RFamides I and II activated sodium channels 164-166. The ability to activate the ENaC/DEG channels by FaLPs were demonstrated for mollusks, annelids, Hydrozoa and partially for mammals. When the invertebrate FaNaC from *Helix aspersa* were expressed in rat hippocampus slice, it was shown that the application of FMRFamide is able to activate the sodium channel current across the cell membrane of neurons and produces large prolonged depolarizations and bursts of action potentials. Nevertheless, mammalian neuropeptides neuropeptide FF and RFRP-1, which have amidated RF terminus, did not affect *Ha*FaNaC-expressing neurons 167. However, the binding of FMRFa or similar RFamides to ASICs has been shown to enhance proton-gated currents 168. To date, there is no evidence that FaLPs can activate the insect ENaC/DEG belonging to pickpocket (PPK) receptors which mediate diverse functions such as the detection of mechano- and chemo-sensory stimuli 164. Of course, that does not mean there is a lack of this activity of FaLPs in insects. It rather shows new perspectives for further studies and a deeper glimpse into the evolutionary history of FaLPs as well as their receptors.

## Conclusions and future directions

Nowadays, it is possible to identify and compare genetic sequences of both neuropeptide precursors and receptors using publicly available datasets. Unfortunately, these sequences do not reveal much about the processed peptides that likely activate the corresponding receptors. In this context, more studies based on mass spectrometry analyses will improve our understanding far more than those based on transcriptome data. However, the greatest limitation is the lack of comprehensive studies on the functions of this group of neuropeptides. Current knowledge about the physiological functions of FaLPs peptides in insects and other arthropods is indeed still discontinuous. Besides the fact that few physiological functions are shared within Pancrustacea (mainly myotropic), there may be species-specific functions in each group (Table 3). To date, significantly less studies have been performed on neurohormonal regulation (also including FaLPs) of physiological processes in crustaceans, so it is extremely challenging to compare the exact physiological properties of these peptides between different arthropod groups. However, the reduced genetic differences in FaLPs genes between crustaceans and insects strongly support the homologous origin of these genes. In Chelicerates and Myriapods, detailed studies on FaLPs are scarce, and it is likely that these peptides perform similar functions in those two groups of arthropods as they do in insects and crustaceans. Nevertheless physiological studies in these groups are needed. This is even more important if FaLPs will be considered, together with other insect neuropeptides, as molecules used to design ecofriendly bioinsecticides 169, 170. To evaluate physiological properties of FaLPs the strategy might involve use factors which affect biostability of peptides, such as additional chemical groups attached to the neuropeptide molecule, e.g., polyethyleneglycol (PEG). So far, several different insect neuropeptide analogues with enhanced biostability have been invented 171-176. For example, PEG-stabilised pyrokinin analogues have been shown to be effective as pest control agents targeting aphids 171.

In insects the exact physiological functions of FaLPs are also not precisely known. So far it is confirmed that FaLPs poses myotropic properties on different visceral muscles and are involved in regulation of cardiac functioning. This is probably due to the fact that historically most of the neuropeptide studies focused on the evaluation of the myotropic activity. However, myoactive properties of FaLPs might indirectly influence other important processes such as reproduction, digestion and feeding. Apart from single studies the exact mechanism in not addressed. Similarly possible direct or indirect (due to myotropic functions) involvement of FaLPs in metabolism needs to checked. This is even more important when we take into account that FaLPs can be involved in the regulation of the circadian clock. In this context also interaction of FaLPs with other neuropeptides should be checked with usage of available techniques such as RNAi or CRISPR/CAS.

Finally, thus far, only one type of receptor, from the GPCR family, has been found in insects and other arthropods. Some studies suggest that FaLPs can also activate the ionotropic receptor 79. So far it has been proved in mollusks, however, it might also be possible in arthropods. It is even more possible if one considers the neuromodulatory and myomodulatory effects of FaLPs, which seem to be highly conserved across different groups of arthropods.

Another unaddressed question is related to evolutionary relations between FaLPs and other RF peptides (especially mammalian RFa peptides). The differences in RFamide peptide gene sequence in mammals and the shared physiological functions with Pancrustacea suggest evolutionary convergence among these gene groups between vertebrates and invertebrates. This convergence may have evolved from a common ancestor of the two lineages. For instance, in tunicates, RFamide peptides have been described and show a conserved role in the regulation of muscle contraction. Therefore, this suggests that, at least for FaLPs peptides, both the genes and the main physiological functions are homologous between invertebrates and vertebrates. At the same time, other physiological functions are likely to evolve because of convergence, and are thus analogous, both within FaLPs and other -RFamide peptides.

## Supplementary Material

Supplementary figures.

## Figures and Tables

**Figure 4:**
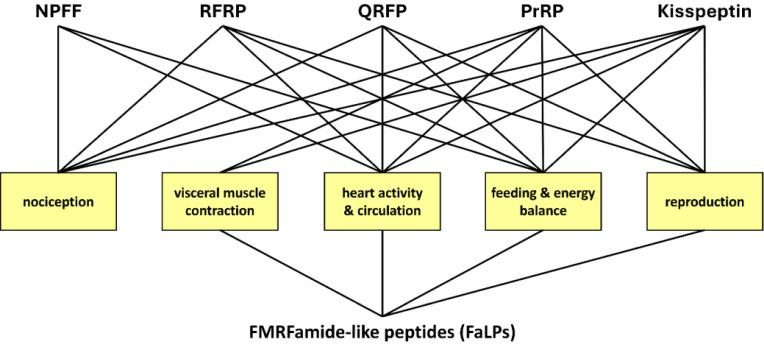
Similar physiological properties of mammalian RFamide peptides (upper row) and insect FaLPs (lower row). FMRFamide-like peptides (FaLPs) are neuropeptides that play a pivotal role in regulating various physiological processes in insects and other arthropods including behaviour, reproduction, and homeostasis. FaLPs mostly act through G-protein coupled receptor and influence muscle activity by modulating Ca^2+^ influx. Historically, the function described for these neuropeptides was primarily associated with myostimulatory activity. After more than three decades of research, it is now well established that FaLPs are implicated in the regulation of circadian rhythms, affecting locomotor activity and phase changes in response to environmental cues. During reproduction, FaLPs influence contractile activity in both the male and female reproductive systems. They also participate in physiological processes such as diapause induction, sleep modulation, and flight regulation in insects. Similarly, in crustaceans, FaLPs regulate the circulatory system, stomatogastric nervous system, and muscle contractions. Nowadays, it is also known how the physiological properties of FaLPs in arthropods share similarities with mammalian RFamide peptides, which are involved in a wide range of functions, including muscle contraction, feeding, reproduction, and stress responses, mediated through various RFamide receptors. Therefore, summarizing the investigated physiological functions in arthropods may be relevant also for future research aiming to test their activity in other organisms such as mammals.

**Table 1:** Examples of amino acid sequences of FaLPs from different arthropod species and humans.

Species	Acronym	Amino acid sequence
*Rhodnius prolixus*	Rhopr-FMRFa	IKDNFIRFa
*Drosophila melanogaster*	Drome-FMRFa-1	SVQDNFMHFa
	Drome-FMRFa-2	DPKQDFMRFa
	Drome-FMRFa-3	TPAEDFMRFa
	Drome-FMRFa-4	SDNFMRFa
	Drome-FMRFa-5	SPKQDFMRFa
	Drome-FMRFa-6	PDNFMRFa
	Drome-FMRFa-7	SAPQDFVRSa
	Drome-FMRFa-8	MDSNFIRFa
*Tenebrio molitor*	Tenmo-FMRFa-1	NNNNFLRFa
	Tenmo-FMRFa-2	SGKTEKNDHFIRFa
	Tenmo-FMRFa-3	SKQDFLRFa
	Tenmo-FMRFa-4	DQHRVVRDRSGNYLRFa
	Tenmo-FMRFa-5	GGSNFMRFa
	Tenmo-FMRFa-6	NSNFLRFa
*Schistocerca gregaria*	Schgr-FMRFa-1	DADAEAAAVDDDGAGGEGDGDGELGLVQTTPRSNFLRLa
	Schgr-FMRFa-2	AGGAHSAFLRLa
	Schgr-FMRFa-3	DRASSGFLRLa
	Schgr-FMRFa-4	GSERNFLRFa
	Schgr-FMRFa-5	SGMADPLTRHDRNFIRFa
	Schgr-FMRFa-6	SGRSGTAQHQAGAEPALWAALLDPRLAVLPAPAGADDADVDGAAGSGRGRVSTRSFLRLa
Periplaneta americana*	Peram-FMRF-1	AEEQNQPPPV
	Peram-FMRF-2	RRCSNRNFVRLa
	Peram-FMRF-3	GHDFDQDDVNSSGEKDESLVRIa
	Peram-FMRF-4	GGRSNDNFIRFa
	Peram-FMRF-5	GGKNDNFIRFa
	Peram-FMRF-6	GGKQDNFIRFa
	Peram-FMRF-7	DRSDNFIRFa
	Peram-FMRF-8	GKTDNFIRFa
	Peram-FMRF-9	GRSDNFIRFa
	Peram-FMRF-10	GKSDNFIRFa
	Peram-FMRF-11	ARPDNFIRFa
	Peram-FMRF-12	GKQDFIRFa
	Peram-FMRF-13	GNSNFVRFa
	Peram-FMRF-15	GGKSGSNFIRFa
	Peram-FMRF-16	ARPSSNFIRLa
	Peram-FMRF-18	GRPSNNFVRFa
	Peram-FMRF-19	pQTEDDDKFVRLS
	Peram-FMRF-20	SGNSNELRRGKL
	Peram-FMRF-21	TDRNFIRLa
	Peram-FMRF-22	SGPSYDEKEQENEDGNSVRLa
	Peram-FMRF-23	SENPSNSRNFIRLa
	Peram-FMRF-24	ALDQNLLVDEHLMRFa
*Carcinus maenas*		RNFLRFa
		pQGNFLRFa
		NRSFLRFa
		SRNYLRFa
		NRNFLRFa
		DRNFLRFa
		APRNFLRFa
		GNRNFLRFa
		APQGNFLRFa
		GAHKNFLRFa
		GLSRNYLRFa
		DGNRNFLRFa
		APQRNFLRFa
		YGNRSFLRFa
		SENRNFLRFa
*Homarus americanus*		GYSDRNYLRF
		NFLRF
		NRNFLRF
		DQNRNFLRF
		GAHKNYLRF
		GNRNFLRF
		GDRNFLRF
		FSHDRNFLRF
		APSKNFLRF
*Daphnia pulex*	Dappu-FIRFa-1	SLRSNFIRFa
	Dappu-FIRFa-2	SALNKNFIRFa
*Tigriopus californicus*		NFLRF
		DYREFVRF
		DDFLRF
		SNDFLRF
*Echinogammarus veneris*		AFPRSFLRFa
		pEPSDLHFIRFa
		AFPVNFSRFa
		pEPSDLNFIRFa
*Latrodectus hesperus*		pQHNIMRFa
		PAGGHNLIHFa
		pEDTSKTPQHSFLYFa
		GHTIMRFa
		DHNMMRFa
		pEGHSMMYFa
		DGHSMLYFa
		GPGHAILSFa
		YGTDTWHSMMNFa
		SENPWDHSTMHFa
		DVDDPWHNMMSFa
		SDILWDHNTMHFa
		SGNPWDQHSTLHFa
		DSHSMIHFa
		NHNLLRFa
		TEGSRKGSHAMIHFa
		SEPWENHNTMHFa
		LTPWESHNTMHFa
		SELWENHNTMHFa
		SEDPWESHNTMHFa
		SDPWENHSTLHFa
*Homo sapiens*	NPFF	SQAFLFQPQRFa
	NPAF	AGEGLNSQFWSLAAPQRa
	RFRP-1	MPHSFANLPLRFa
	RFRP-3	VPNLPQRFa
	QRFP43	EDEGSEATGFLPAAGEKTSGPLGNLAEELNGYSRKKGGFSFRFa
	QRFP26	TSGPLGNLAEELNGYSRKKGGFSFRFa
	PrRP31	SRTHRHSMEIRTPDINPAWYASRGIRPVGRFa
	PrRP20	TPDINPAWYASRGIRPVGRFa
	Kisspeptin-54	GTSLSPPPESSGSPQQPGLSAPHSRQIPAPQGAVLVQREKDLPNYNWNSFGLRa
	Kisspeptin-10	YNWNSFGLRFa

To maintain consistency with the existing databases the nomenclature of single analogue has been retain (FMRFa instead of FaLPs). Note the *C*-terminal typical F-L/M/I/V-R-L/Fa sequence. * List according to 44, note Peram-FMRF-1, 19, 20 are not amidated

**Table 2:** Summary of known physiological properties of FaLPs in crustaceans

Function	Neuropeptide	Species	References
**Cardiotropic effects:**			
positive chronotropic and negative inotropic effects on heart	TNRNFLRFa (F1) SDRNFLRFa (F2)	*Carcinus means*	130
increase in the rate and amplitude of heartbeats	TNRNFLRFa (F1) SDRNFLRFa (F2)	*Procambarus clarkii*	131
	DRNFLRFa (DF2) NRNFLRFa (NF1)	*Homarus americanus*	51, 136
cardioexcitation in semi-isolated heart and cardioinhibition *in vivo*	TNRNFLRFa (F1) SDRNFLRFa (F2)	*Cancer magister*	132, 133
increase in the rate and strength of the heartbeat	TNRNFLRFa (F1)	*Homarus americanus*	134
increase in the amplitude of electrically evoked contractions of ostial valve muscle	SDRNFLRFa (F2)	*Homarus americanus*	135
dose-dependent increase of heart rate	GYNRSFLRFa	*Callinectes sapidus*	48
chrono- and inotropic positive effects	TNRNFLRFa (F1) SDRNFLRFa (F2) GYNRSFLRFa	*Callinectes sapidus*	137
increase the frequency of bursts and the number of spikes per burst	GAHKNYLRFa	*Cancer borealis*	121
**Motility of the digestive system:**			
activation of gastric motor patterns	TNRNFLRFa (F1) SDRNFLRFa (F2)	*Cancer borealis*	50
activation of pyloric and gastric mill	TNRNFLRFa (F1) SDRNFLRFa (F2)	*Cancer borealis*	50, 139, 142
		*Homarus americanus*	140
		*Procambarus clarkii*	141
**Myotropic effects:**			
increase in nerve-evoked tension and increase of postsynaptic potentials	TNRNFLRFa (F1) SDRNFLRFa (F2)	*Procambarus clarkii*	143
rhythmic depolarizations and contractions	YGGFMRFa	*Palaemon serratus*	144
tonic contractions of muscle without nerve activation	TNRNFLRFa (F1)	*Homarus americanus*	134
increase of amplitude of excitatory junctional potentials	NRNFLRFa DRNFLRFa	*Procambarus clarkii*	136

**Table 3:**
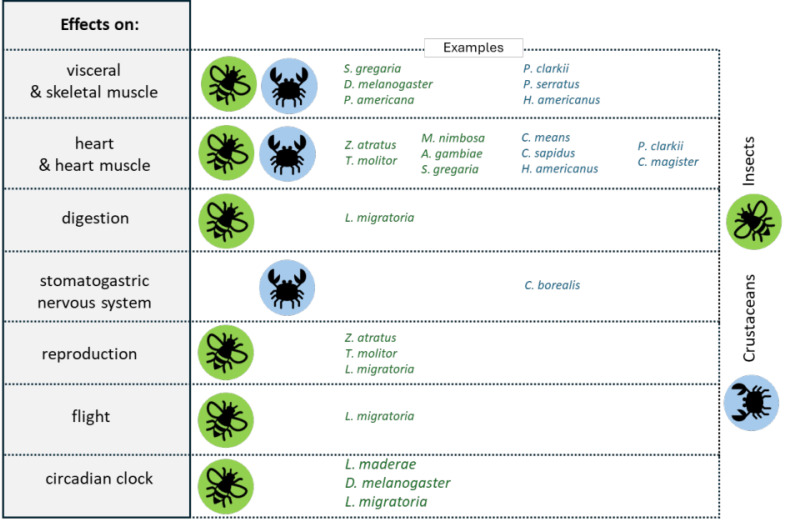
Comparison of known physiological properties of FaLPs in insects and crustaceans
