# Supplementary material for: FMRFamide-like peptides (FaLPs) - an overview of diverse physiological roles in insects and other arthropods
Source: Int J Biol Sci. 2025 Mar 31;21(6):2725–46. doi: 10.7150/ijbs.106382 (PMC12035899; doi:10.7150/ijbs.106382)
Supplement: Supplementary file 1 — Supplementary figures. [file ijbsv21p2725s1.pdf]

> *Limulus polyphemus* XP_013783533.2 predicted

MLLPPVRTSPTKLLVFILCHQMGFCFNHLFQPLDTEMNHIQDLKIQHVLDNTAHAEENDVQRRIFGNSFNDEILSNQHQFPNDRPDSNTLSNVYQYPKLQMLTNNFF
PQGFYQVPLKENMVNVHYEEAKKVKRQSPDNVFIRFGRNTESTSKYSSDVTSNLLFTLLNFYFPFNLQTMEKLRTSDSFGKHNQEKYYLSNLLKVKVLDTFRNNKQRS
GKAKPLALQTRNGNALVRFGRSVKEYSDIGYDSERGISSTTLNKLDFDAIDDQNLRTSHSVQPDYEFLSKNYVSNKFQKNYNTLPFIFLSCIKGLEKLATIGDHKDIPRYT
RKVSRNVICQRPIEKCGLFSDSNHEILGPFSEKKLHKRSTNSDITFDRNPISMTRFRRSPNAVLRSGKTTKSVVRFGRKPNSVVTFRKNVIPLFKFNKLINSAARFDKTDS
SLNRFGRADNSILRFGRSPSSMIRFGRAPTSMIRFGRSPSSVVRFGRAPTSMIRFGRSPSSVMRFRRAPTSMIRFGRSPSSVMRFRRAPTSMIRFGRSPSSVMRFGRAP
TSMIRFGRSPSSVVRFGRAPTSMIRFGRSPSSMVRFGRALTSMIRFGRAPNFMNRTDGTPISMTRFRRTPNSMIRFGRTPSSMIKFGRTPTSIIRNGRIPNSMIRFGRTQS
SVKRLQKTSDSSIRSVGFGRVPRSTIRFGRAPNSKVNFGRNLDESIKSRKKSNAKMSVHNEEINNKFIGYLTTPRTLENFNEIIKLTDRYGCLSDKKCVDVLKAERNNDHF
YENSAPDSLQNTAFYKNGNLKHFATKEKNNKNTPSVKSITEKFNLMSP

> *Ixodes scapularis* Isca_Trinity_c85671_g1_i1

MKFALLLCLLCWLENPLPSCTGNHVQQGEPESVNEPKQSAQPLDRVARTADPDNAKDIGGRDPYSDYNTLMALMGPRRHRYLHFGRKRALPLYSDVPVDQVEGSD
DYIGDDYDASSAESLEDAMRWAKPYLGDGLHGDVVLSGALEDGQVIRYKRDVSMASVRDDELDTNTDQKRRALIEVHDELARDGNAGPYLDWQGREKKSQNRILH
FGKREGQHESTQLGSDEVQGDIKRAMNRILHFGKRVRDDATSDFQGDDYGWYSSGDKRATNRIMHFGKRQPEEILISDESSGPQIQIHGNDKKSINRILHFGKREGN
EAFDTDLVESGYRKRNARSNRIMHFGKRTDGGLTSDFDPESHTALKRATNRIMHFGKRESALSSSLEDQLKRDFFEWKKRYTNRMLYFGKKRPQDRYTDKRITNRIMH
FGKRGVIFPLSDETDDSSGKQKRQLKNSILHFGKRDDEKSIEKRTRNRIMHFGKREEGYPYENRLASDKHLGDRILHFGKQEPHHQAADFLNKRSTANADLQFDNED
NGSPYLVDKKITNRILHFGKRLDDSAEDPGKVSGKPKQHVSSVNSDIKFEDSFLFEEHKPHNRRKRSLGFDQYDLDETLERVVHQLMDAGYPKRVALGHPGIPGHLH
LPHAFVAAHVYGSELPRMLSRPSRSDRFFPVPYSGEHREAPKGPSRNVFLRFG

> *Centruroides sculpturatus* XP_023234646.1 FMRF-amide neuropeptides-like

MLFKYSMSLFNLAVLLVVLGVEANTDENENAPANEFFDNYPDNAKRSHNIMRFGKRMIDDELDQKRNHNMIHFGKRAFSISGDNRFPELLRILRSDDRDWWKRGH
NIMRFGKRADDHHFIHFGKRDDEDATSNEYYDNIDDGDDHTMLKRPQHALIRFGKRFLFPRPSKSEESPNMGNKRFPHTMIHFGKRDYEDQEYSSNYSNDNEDMV
DSEEDLLPMNSEKRAHSMIHFGKKDEPYELDDKRSHQLMHFGKRYDGDEEKRDHKLIHFGKRFELEDDKRAHQLIHFGKRLDDEEEKRSHSLMHFGKRLNDEEEE
KRSHSLMHFGKRLNDEDEKRSHSLMHFGKRFNTEFDKRVHSMIHFGKRMDGDMDKRGDYKLLFYPKKEDNHHHNIMHFGKRNAESMNDEINQGPKGETSRTKRS
SKKEDISKNKVTTHTSSIDTPFNKSQSKKND

> *Hanseniella nivea* GERZ01043487.1

MAVIGGLTLVTLFLIGRVAGDECFQPIETHGTSAPPHVTTPTSRDHSLNQSPTSSKTKCAGKSEASVTLEKRSLNKNFLRFGRGYDDVVEDATNEDGELSDVMEKKSAL
NKNFLRFGRPSAPLYDDDLKRGAPLDRNFLRFGRTAPYYDEGVKRGAPLDRNFLRFGRTAPLYDEGVKRGAPLDRNFLRFGRTPADMTSEKRGAALDRNFLRFGRAR
SVDIRKLLDDYYSKRGALDRNFLRFGRSSPSPGPEVSSESNPASAEEEEGFPEMKRSKLQKNFLRFGRGPVDYYWNYDEGAASDKRSALNKNFLRFGRSVDYSRSH

Supplementary material 1. Examples of neuropeptide precursors in chosen arthropods. Blue, signal peptide; yellow, predicted sequence of bioactive neuropeptide; green, predicted C-terminal glycine amidation site; red, predicted cleavage sites of neuropeptides.

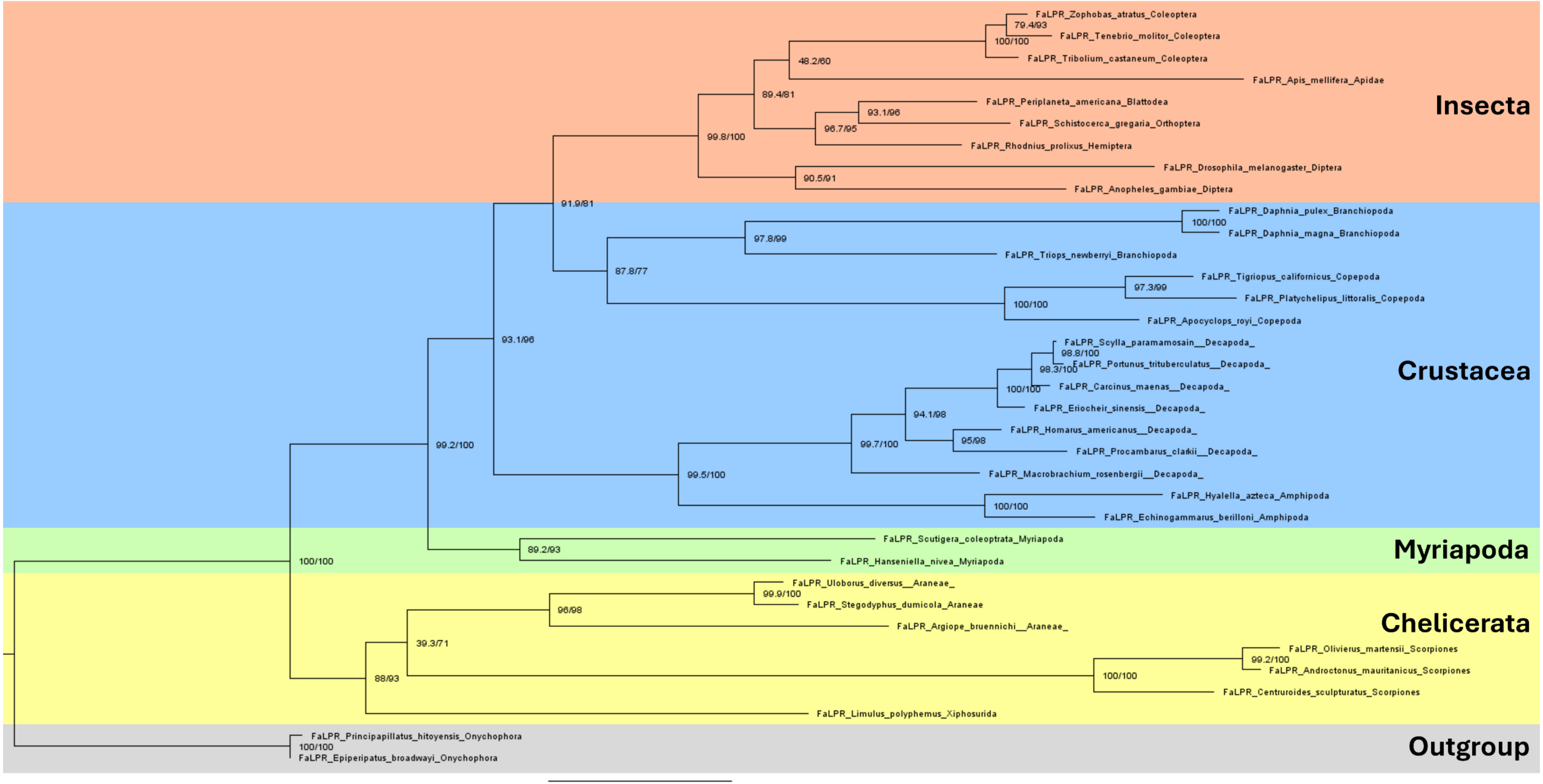

Supplementary material 2. Phylogenetic tree of FaLPs receptors of different arthropod species. FASTA files of aligned receptor sequences were converted into PHYLIP format using AliView 1.18-beta7 [85]. Best-fit substitution models for subsequent phylogenetic analyses were predicted with ModelFinder [86-88], implemented in IQ-TREE release 2.1.4b [89]. All phylogenetic analyses were rooted using the Onychophora. Maximum likelihood (ML) analyses were carried out using IQ-TREE 2.1.4b. ML analyses were evaluated with 1,000 ultra-fast bootstraps (UFBoot) [90] and the Shimodaira–Hasegawa-like approximate likelihood ratio test (SH-aLRT) [91]. Tree was visualized using FigTree 1.4.2 (http://tree.bio.ed.ac.uk/). *Zophobas morio* XM_064053713; *Tenebrio molitor* XM_069053802.1; *Tribolium castaneum* XM_064355982; *Apis mellifera* NM_001327961; *Drosophila melanogaster* BK000442.1; *Anopheles arabiensis* XM_040299364; *Periplaneta americana* XM_069833400; *Schistocerca gregaria* XM_049991159; *Rhodnius prolixus* GECK01020270; *Triops newberryi* GEHY01003975; *Daphnia pulex* XM_046606704; *Daphnia magna* XM_032938373; *Tigriopus californicus* XM_059238139; *Platychelipus_littoralis* GHXK01071804; *Apocyclops_royi* GHAJ01046269; *Carcinus maenas* GFYW01107394; *Scylla_paramamosain* XM_064011162; *Portunus_trituberculatus* XM_045272217; *Eriocheir sinensis* XM_050878336; *Homarus americanus* XM_042367317; *Procambarus clarkii* XM_069327162; *Macrobrachium rosenbergii* XM_067094238; *Hyalella azteca* XM_018170252; *Echinogammarus_berilloni* GHCU01057970; *Scutigera coleoptrata* GCAQ01015165; *Hanseniella nivea* GERZ01045459; *Uloborus diversus* XM_054866386; *Stegodyphus dumicola* XM_035353751; *Argiope bruennichi* XM_056094528; *Centruroides sculpturatus* XM_023384283; *Olivierus martensii* AYEL01080239; *Androctonus mauritanicus* WUQG02432180; *Limulus polyphemus* XM_022384870; *Principapillatus hitoyensis* GKOM01018087; *Epiperipatus broadwayi* JAQFVV010006202.
